# Indobufen Versus Aspirin After CABG for Unstable Angina in Patients Aged 70 Years and Older

**DOI:** 10.3390/jcdd13080356

**Published:** 2026-07-29

**Authors:** Ben Huang, Yubin Zhong, Libin Yang, Xin He, Quanlin Yang, Yongxin Sun, Chunsheng Wang

**Affiliations:** 1 Department of Cardiac Surgery, Zhongshan Hospital, Fudan University, Shanghai 200032, China; huang.ben@zs-hospital.sh.cn (B.H.); 24111210176@m.fudan.edu.cn (L.Y.); 2 Department of Cardiac Surgery, Zhongshan Hospital Xiamen Branch, Fudan University, Xiamen 361006, China; zhong.yubin@zsxmhospital.com (Y.Z.); yang.quanlin@zsxmhospital.com (Q.Y.); 3 Shengli Clinical Medical College, Fujian Medical University, Fuzhou 350001, China; 1241120254@fjmu.edu.cn

**Keywords:** unstable angina, coronary artery bypass grafting, discharge antiplatelet pathway, indobufen, aspirin, inverse probability of treatment weighting, graft surveillance

## Abstract

Background: Postoperative antiplatelet selection after coronary artery bypass grafting (CABG) in adults aged ≥70 years requires balancing ischemic protection against bleeding risk. Methods: We conducted a single-center retrospective cohort study of adults aged ≥70 years who underwent isolated CABG for unstable angina between 2017 and 2021. The exposure was the initial discharge antiplatelet pathway: aspirin 100 mg once daily or indobufen 100 mg twice daily as the cyclooxygenase-inhibitor component, combined with a P2Y12 inhibitor during the first postoperative year and followed by the same cyclooxygenase inhibitor during the second year. Propensity scores were estimated using multivariable logistic regression with 16 prespecified baseline covariates, and stabilized inverse probability of treatment weighting (IPTW) was applied. The primary analyses used IPTW-weighted Cox models. Results: Among 336 screened patients, 79 were included in the final analytic cohort (aspirin-based pathway, *n* = 49; indobufen-based pathway, *n* = 30). After weighting, measured covariate balance improved, although residual imbalance remained for prior myocardial infarction and prior percutaneous coronary intervention. At 24 months, no statistically significant association was observed between pathway and MACCE (HR 0.858, 95% CI 0.257–2.866; *p* = 0.803), BARC grade ≥2 bleeding (HR 0.359, 95% CI 0.093–1.385; *p* = 0.137), or NACE (HR 0.526, 95% CI 0.204–1.360; *p* = 0.185). Graft surveillance was evaluable in 77/79 patients at 1 year and 71/79 at 2 years and was analyzed descriptively. Conclusions: In this real-world cohort of older patients undergoing CABG for unstable angina, an initial indobufen-based discharge pathway was not statistically significantly associated with 24-month MACCE, BARC grade ≥2 bleeding, or NACE compared with an aspirin-based pathway after stabilized IPTW. These findings add exploratory CABG-specific data on indobufen in older patients and should be confirmed in larger prospective studies.

## 1. Introduction

Coronary artery bypass grafting (CABG) remains a standard revascularization strategy for patients with complex coronary anatomy, multivessel disease, or left main coronary involvement [1]. The clinical benefits of CABG depend not only on surgical success but also on sustained graft patency and effective secondary prevention [2,3,4]. However, uncertainty persists about how postoperative antiplatelet therapy should be individualized in adults aged ≥70 years, particularly when treatment is delivered as a staged approach rather than a fixed regimen. Aspirin has long been considered the cornerstone of postoperative antiplatelet therapy after CABG, whereas dual antiplatelet therapy (DAPT) is used more selectively, especially after acute coronary syndromes and in selected off pump CABG settings [1,4,5,6]. Most contemporary studies of antiplatelet therapy after CABG have focused on ticagrelor-based strategies, saphenous vein graft patency, or mixed age populations that include patients with acute coronary syndrome [7,8,9]. Consequently, available data provide limited guidance for adults aged ≥70 years with unstable angina [10,11,12]. This gap is clinically important because older adults often have multimorbidity, polypharmacy, reduced renal reserve, prior gastrointestinal disease, baseline anemia, and lower tolerance for treatment interruption related to bleeding [13,14,15]. In such patients, the clinical question extends beyond ischemic protection alone to whether a discharge strategy is sufficiently tolerable to be maintained through both the initial DAPT phase and the subsequent maintenance phase [13,14,15,16]. Indobufen is a reversible cyclooxygenase inhibitor that suppresses thromboxane-mediated platelet activation and permits recovery of platelet function after discontinuation [17,18]. This pharmacodynamic profile may be clinically relevant when temporary interruption of antiplatelet therapy is required during follow-up, including evaluation of bleeding symptoms or noncardiac procedures.

Systematic reviews have summarized the clinical evidence for indobufen in coronary artery disease, but most available data remain outside postoperative CABG populations [19,20]. Indobufen has been used in East Asia, particularly in patients with aspirin intolerance or gastrointestinal concerns, and recent studies from China have expanded the clinical evidence base for indobufen-based antiplatelet strategies. In the OPTION trial, indobufen plus clopidogrel was evaluated as an aspirin-replacement DAPT strategy after coronary drug-eluting stent implantation [21]. The ASPIRATION registry further assessed indobufen-based DAPT in a real-world Chinese PCI population [22]. More recently, indobufen has also been evaluated in elderly patients with acute coronary syndrome after PCI [23]. However, PCI evidence cannot be directly extrapolated to postoperative CABG care. CABG-specific evidence remains limited, with available data including an earlier graft-patency study, a Chinese CABG randomised study, and a recent multicenter retrospective post-CABG cohort [24,25,26]. Therefore, further CABG-specific evidence in older postoperative patients treated with staged discharge pathways remains needed.

Therefore, we conducted a retrospective study of adults aged ≥70 years who underwent isolated CABG for unstable angina. Rather than comparing two abstract DAPT regimens, we evaluated two initial discharge antiplatelet pathways: first-year dual antiplatelet therapy anchored by either aspirin or indobufen, followed by second-year maintenance with the same cyclooxygenase inhibitor. We aimed to estimate exploratory 24-month clinical, bleeding, and graft-surveillance outcomes associated with these two staged discharge pathways in an older postoperative CABG cohort.

## 2. Materials and Methods

### 2.1. Study Design and Source Population

This single center, retrospective cohort study used an institutional cardiac surgery registry and medical records to identify patients aged ≥70 years who underwent isolated CABG for unstable angina between 1 January 2017, and 31 December 2021. The inclusion period ended in December 2021 to allow the possibility of 24-month outcome ascertainment before data extraction. The protocol was approved by the Ethics Committee of Zhongshan Hospital, Fudan University (approval number B2025-377), with waiver of informed consent because of the retrospective design.

### 2.2. Exposure Definition: Discharge Antiplatelet Pathway

The exposure of interest was the initial discharge antiplatelet pathway. An aspirin-based pathway was defined as aspirin 100 mg once daily plus a P2Y12 inhibitor during the first postoperative year, followed by aspirin monotherapy during the second year. An indobufen-based pathway was defined as indobufen 100 mg twice daily plus a P2Y12 inhibitor during the first postoperative year, followed by indobufen monotherapy during the second year. No weight- or renal-function-based dose reduction was identified in the available records. Treatment was determined by treating physicians in routine practice. The specific determinants of pathway selection were not systematically recorded, and residual confounding by indication cannot be excluded. The primary analysis classified patients according to the initial discharge pathway. Documented pathway modifications during follow-up were summarized separately and addressed in a sensitivity analysis censoring patients at the time of modification.

### 2.3. Eligibility Criteria

Patients were eligible if they underwent isolated CABG for unstable angina, were aged 70 years or older, were discharged on one of the two prespecified antiplatelet pathways, and had sufficient data for clinical outcome ascertainment and propensity-score estimation. Exclusion criteria were redo CABG, concomitant cardiac procedures, oral anticoagulation or other nonstudy discharge regimens, absence of linkable 24-month clinical follow-up, and missing baseline variables required for the weighting model. The absence of annual graft imaging alone was not an exclusion criterion for the clinical analyses.

### 2.4. Outcome Definitions

The main clinical effectiveness outcome was 24-month major adverse cardiovascular and cerebrovascular events (MACCE), defined as all cause death, nonfatal myocardial infarction, ischemic stroke, or clinically driven repeat revascularization. Additional clinical outcomes comprised the individual MACCE components analyzed separately. Myocardial infarction events were identified from clinical records using available diagnostic documentation; perioperative biomarker-only events without clinical adjudication as follow-up myocardial infarction were not counted as nonfatal myocardial infarction outcomes. Safety was assessed using Bleeding Academic Research Consortium (BARC) grade ≥2 bleeding [27]; BARC grade ≥3 bleeding was summarized descriptively. Net adverse clinical events (NACE) were defined as MACCE or BARC grade ≥2 bleeding. Deaths were also categorized as cardiovascular or non-cardiovascular when ascertainable from records. Clinical outcomes were ascertained by retrospective review of medical records and follow-up documentation. Graft imaging was performed during clinical follow-up and was not mandated by the study protocol. Therefore, graft findings were summarized descriptively among patients with evaluable imaging at each time point, rather than analyzed as comparative patency endpoints. Composite graft occlusion was defined at the patient level as any graft occlusion on annual angiography or computed tomography angiography. Left internal mammary artery (LIMA) and saphenous vein graft (SVG) occlusion were also summarized separately. Occlusion was defined as absent contrast filling, abrupt interruption of contrast flow, or a thread-like graft appearance. Graft imaging information was obtained from institutional imaging records and, when available, from external coronary CTA or angiography reports and images provided by patients or accessible through cloud-based imaging platforms during routine follow-up.

### 2.5. Statistical Analysis

Continuous variables are presented as mean with standard deviation or median with interquartile range, and categorical variables are presented as counts and percentages. Baseline characteristics were summarized descriptively. Because treatment allocation was not randomized, balance between the initial aspirin-based and indobufen-based discharge pathways was assessed using absolute standardized mean differences rather than significance testing.

Clinical outcomes were analyzed according to the initial discharge antiplatelet pathway. Time-to-event outcomes were evaluated using Cox proportional hazards models and are reported as hazard ratios with 95% confidence intervals. IPTW-weighted Kaplan–Meier curves are shown for the main clinical outcomes, with numbers at risk reported as unweighted counts. Graft-surveillance findings were summarized descriptively among patients with evaluable imaging at each time point and were not analyzed as time-to-event outcomes.

Propensity scores for the initial indobufen-based discharge pathway were estimated using multivariable logistic regression. Covariates were prespecified on clinical grounds to reflect factors related to treatment selection or outcome risk, including demographics, ischemic and bleeding history, gastrointestinal protection, perioperative bleeding, laboratory profile, cardiac function, operative burden, and concomitant P2Y12 inhibitor use. The model included age, sex, prior myocardial infarction, prior percutaneous coronary intervention, prior gastrointestinal bleeding, prior stroke, diabetes mellitus, current smoking, proton-pump inhibitor use, early postoperative bleeding, hemoglobin, serum creatinine, albumin, left ventricular ejection fraction, number of grafts, and P2Y12 inhibitor type. These variables were complete in the final analytic cohort, and no imputation was performed.

The primary analysis used stabilized inverse probability of treatment weights for the average treatment effect, without weight truncation. Balance after weighting was assessed using absolute standardized mean differences, propensity-score overlap, weight distribution, and effective sample size. Sensitivity analyses included truncating stabilized weights at the 5th and 95th percentiles, additional adjustment for residual imbalance after weighting, and censoring at documented treatment-pathway modification.

All tests were two-sided. *p*-values were reported descriptively, with interpretation focused on effect estimates and 95% confidence intervals because of the small sample size and limited number of events. Analyses were performed using R software (version 4.5.2; R Foundation for Statistical Computing, Vienna, Austria).

## 3. Results

### 3.1. Study Cohort

**Figure 1:**
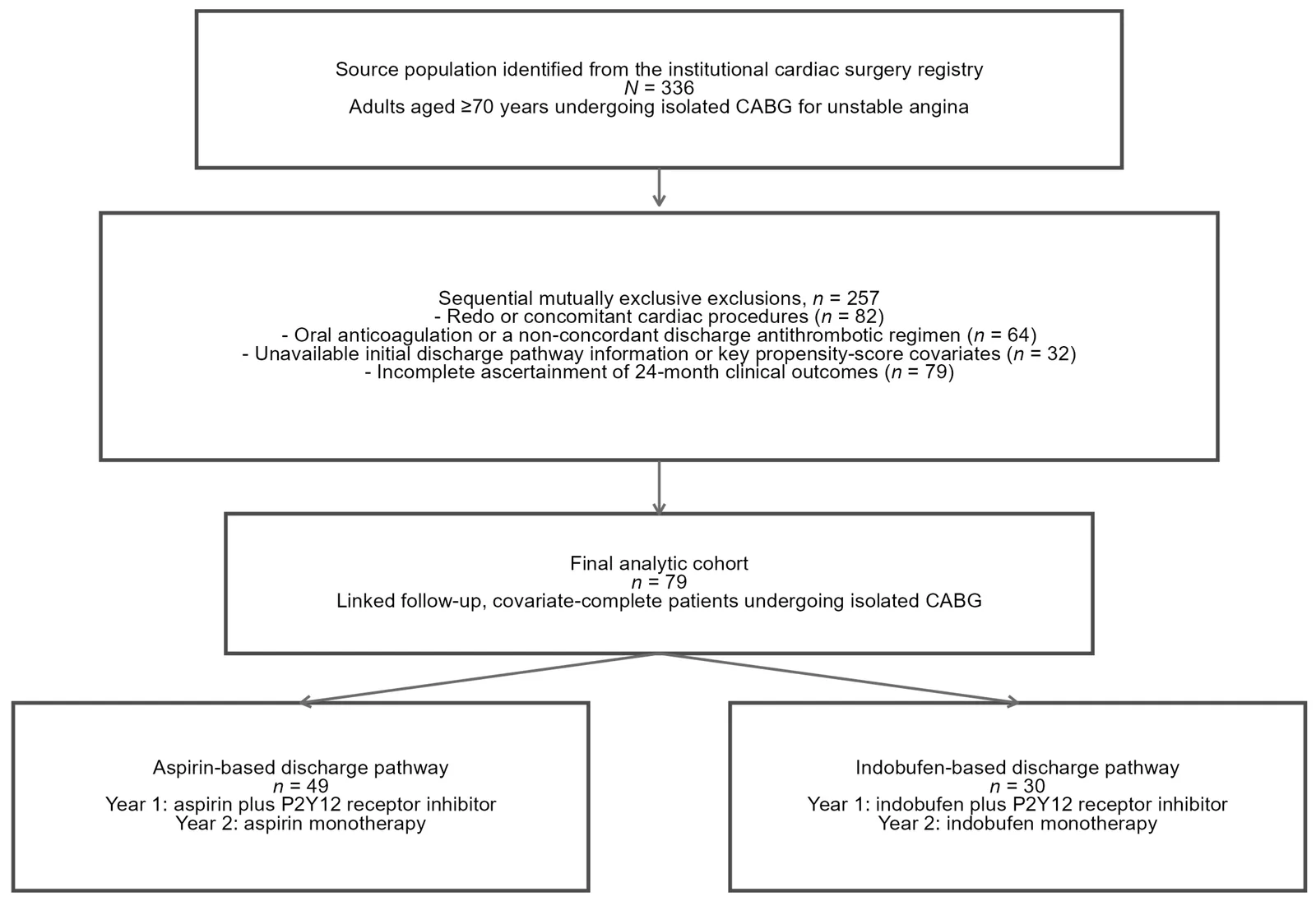
Patient screening flow diagram. Exclusion categories were applied sequentially and were mutually exclusive. CABG, coronary artery bypass grafting; PS, propensity score.

Of 336 screened patients aged ≥70 years who underwent isolated CABG for unstable angina, 257 were excluded sequentially as follows: redo or concomitant cardiac procedures (*n* = 82), oral anticoagulation or a non-concordant discharge antithrombotic regimen (*n* = 64), unavailable initial discharge pathway information or key propensity-score covariates (*n* = 32), and incomplete ascertainment of 24-month clinical outcomes (*n* = 79). The final analytic cohort included 79 patients: 49 in the aspirin-based pathway and 30 in the indobufen-based pathway (Figure 1). Available baseline characteristics of patients included in the final analytic cohort and those excluded because of incomplete ascertainment of 24-month clinical outcomes are summarized in Supplementary Table S1. Overall, the baseline characteristics were comparable between included and excluded patients, with most standardized mean differences (SMDs) below 0.20. Age (SMD = 0.265), left ventricular ejection fraction (SMD = 0.247), and baseline anemia (SMD = 0.256) showed modest imbalances, suggesting potential but limited selection bias related to outcome ascertainment. Detailed reasons for exclusion due to unavailable initial discharge pathway information and/or propensity-score covariates are provided in Supplementary Table S2.

### 3.2. Baseline Characteristics and IPTW Diagnostics

**Figure 2:**
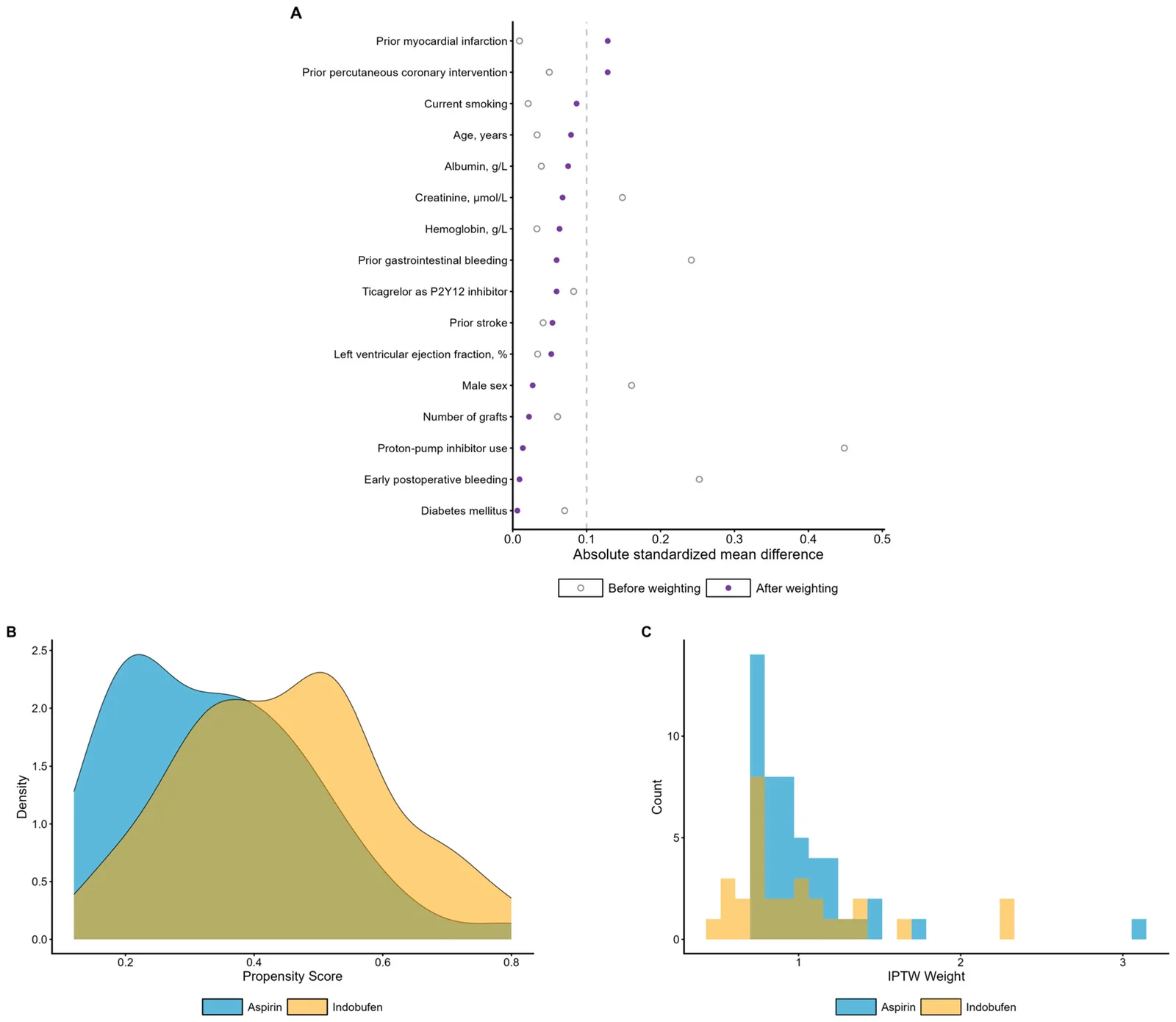
Propensity-score and inverse probability of treatment weighting diagnostics. (**A**), Absolute standardized mean differences before and after IPTW based on a propensity-score model including 16 prespecified baseline covariates. The dashed line denotes an absolute standardized mean difference of 0.10. (**B**), Propensity score overlap by discharge antiplatelet pathway. (**C**), Distribution of stabilized IPTW weights. Residual imbalance remained for prior myocardial infarction and prior percutaneous coronary intervention. IPTW, inverse probability of treatment weighting; SMD, standardized mean difference.

Baseline characteristics and covariate balance are shown in Table 1. After stabilized IPTW, overall covariate balance improved substantially. Residual imbalance remained for prior myocardial infarction (weighted absolute SMD = 0.129) and prior percutaneous coronary intervention (weighted absolute SMD = 0.128). Propensity-score overlap, weighted balance, and weight distributions are shown in Figure 2.

### 3.3. Treatment Details and Pathway Modification

The documented pathway doses were aspirin 100 mg once daily and indobufen 100 mg twice daily. Detailed antiplatelet treatment characteristics are summarized in Supplementary Table S4A. Clopidogrel was the background P2Y12 inhibitor in most patients, with ticagrelor used in the remainder. Seven documented pathway modifications occurred during follow-up: four from aspirin to indobufen at 110, 188, 274, and 402 days, and three from indobufen to aspirin at 96, 221, and 365 days. These modifications and their documented reasons are summarized in Supplementary Table S4B. These data describe documented changes in the cyclooxygenase-inhibitor component and do not fully capture temporary interruptions or day-to-day adherence.

### 3.4. Twenty-Four-Month Clinical Outcomes

**Figure 3:**
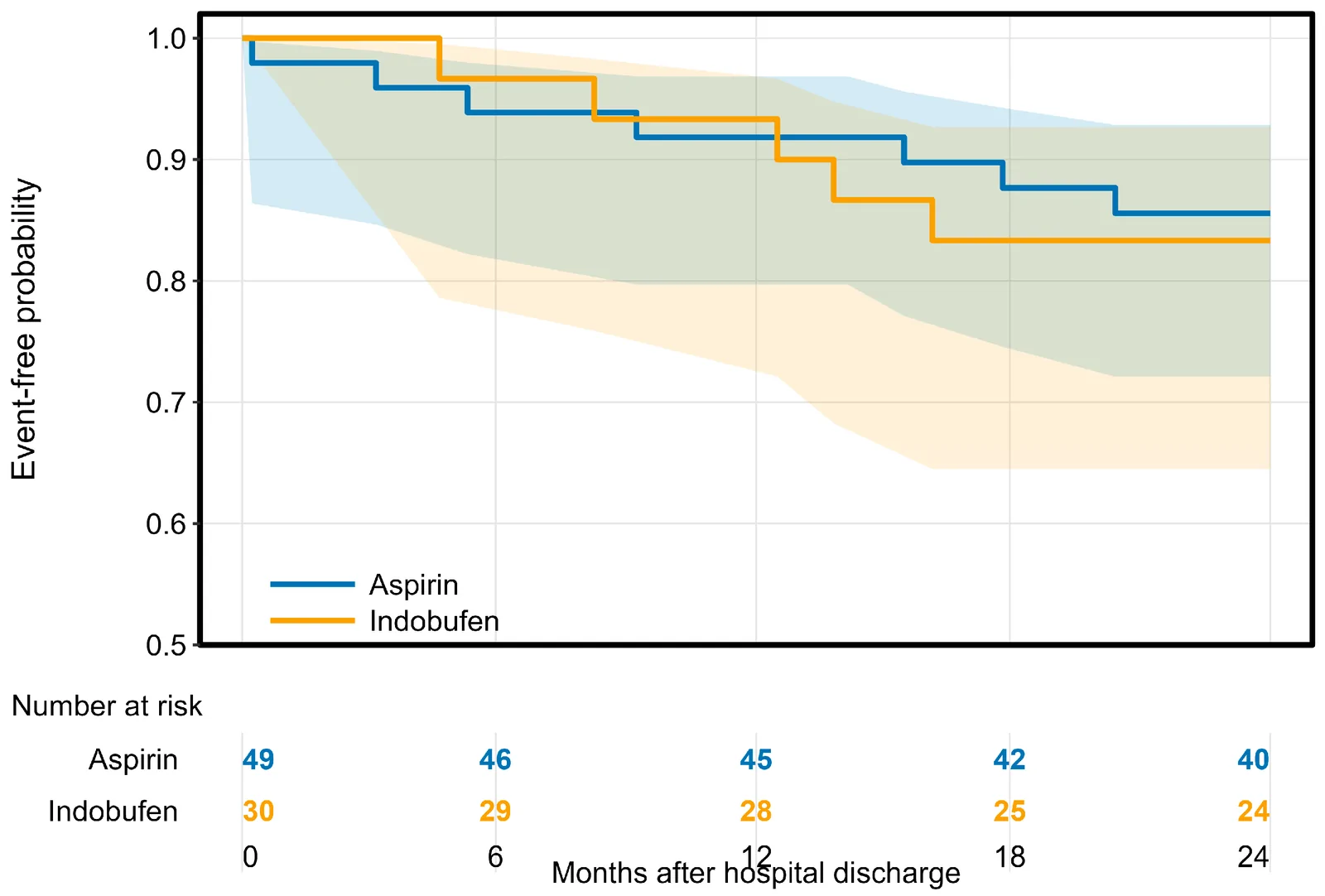
IPTW-weighted Kaplan–Meier curve for MACCE-free survival. Shaded areas indicate 95% confidence intervals (blue: aspirin; orange: indobufen). Numbers at risk are shown as unweighted counts. MACCE, major adverse cardiovascular and cerebrovascular events; IPTW, inverse probability of treatment weighting.

**Figure 4:**
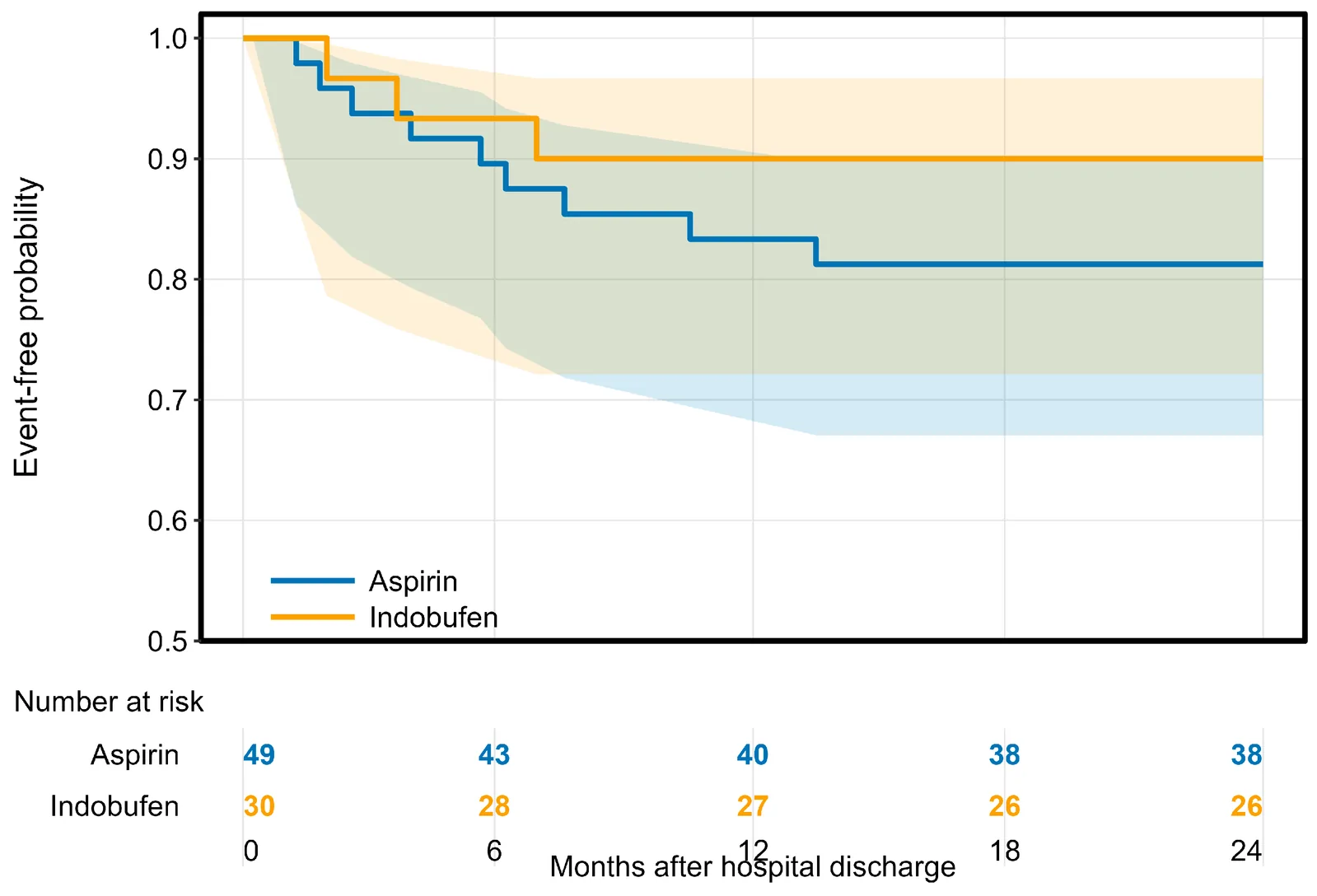
IPTW-weighted Kaplan–Meier curve for BARC grade ≥2 bleeding-free survival. Shaded areas indicate 95% confidence intervals (blue: aspirin; orange: indobufen). Numbers at risk are shown as unweighted counts. BARC, Bleeding Academic Research Consortium; IPTW, inverse probability of treatment weighting.

**Figure 5:**
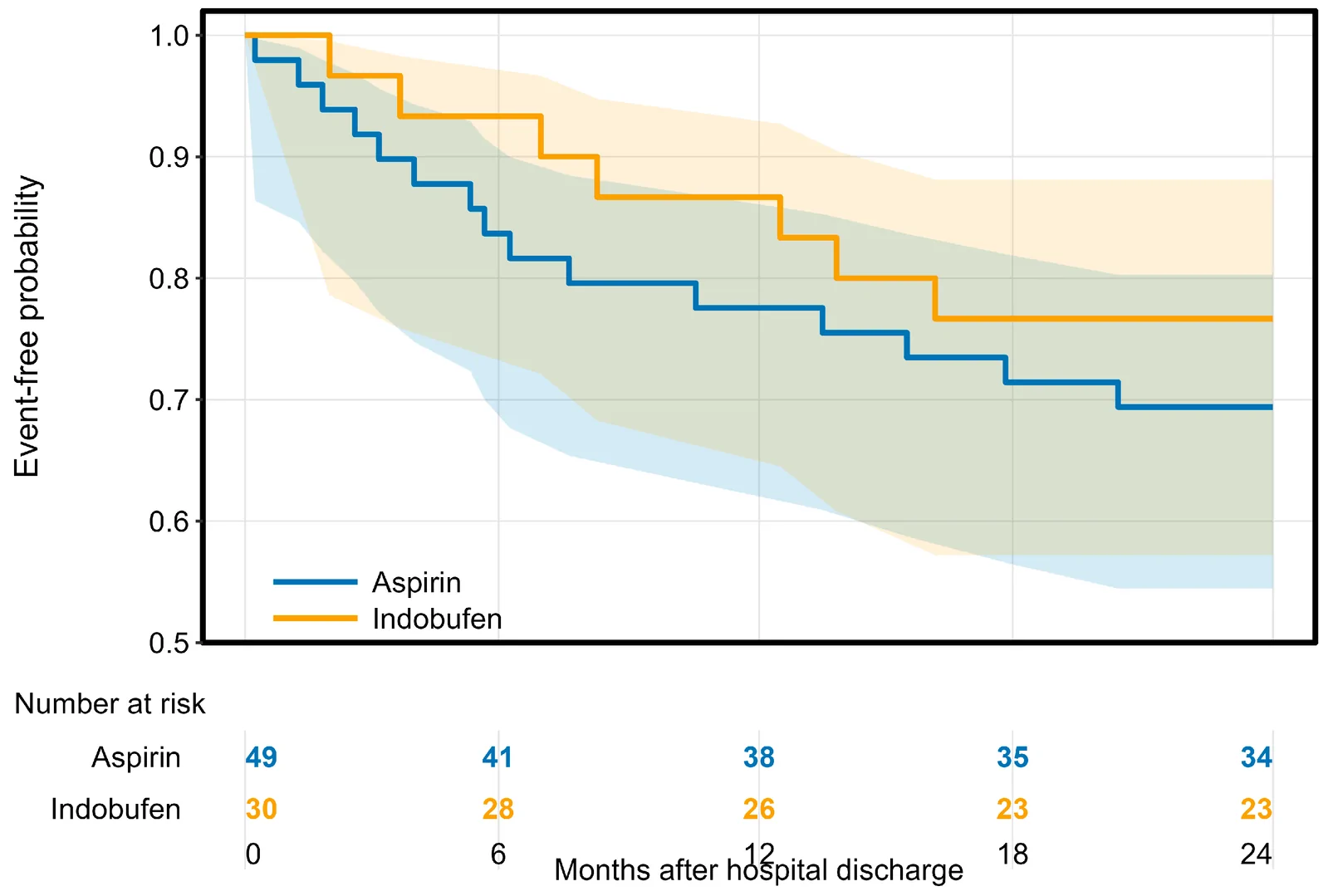
IPTW-weighted Kaplan–Meier curve for NACE-free survival. Shaded areas indicate 95% confidence intervals (blue: aspirin; orange: indobufen). Numbers at risk are shown as unweighted counts. NACE, net adverse clinical events; IPTW, inverse probability of treatment weighting.

Twenty-four-month clinical outcomes are summarized in Table 2 and Figure 3, Figure 4 and Figure 5. In IPTW-weighted Cox models, no statistically significant association was observed between the initial discharge pathway and MACCE (HR 0.858, 95% CI 0.257–2.866; *p* = 0.803), BARC grade ≥2 bleeding (HR 0.359, 95% CI 0.093–1.385; *p* = 0.137), or NACE (HR 0.526, 95% CI 0.204–1.360; *p* = 0.185). Estimates were imprecise and confidence intervals were wide. The clopidogrel-only sensitivity analysis is summarized in Supplementary Table S5. Additional sensitivity analyses, including weight truncation, additional adjustment for residual imbalance, and censoring at documented pathway modification, are summarized in Supplementary Table S6.

### 3.5. Descriptive Graft-Surveillance Outcomes

**Figure 6:**
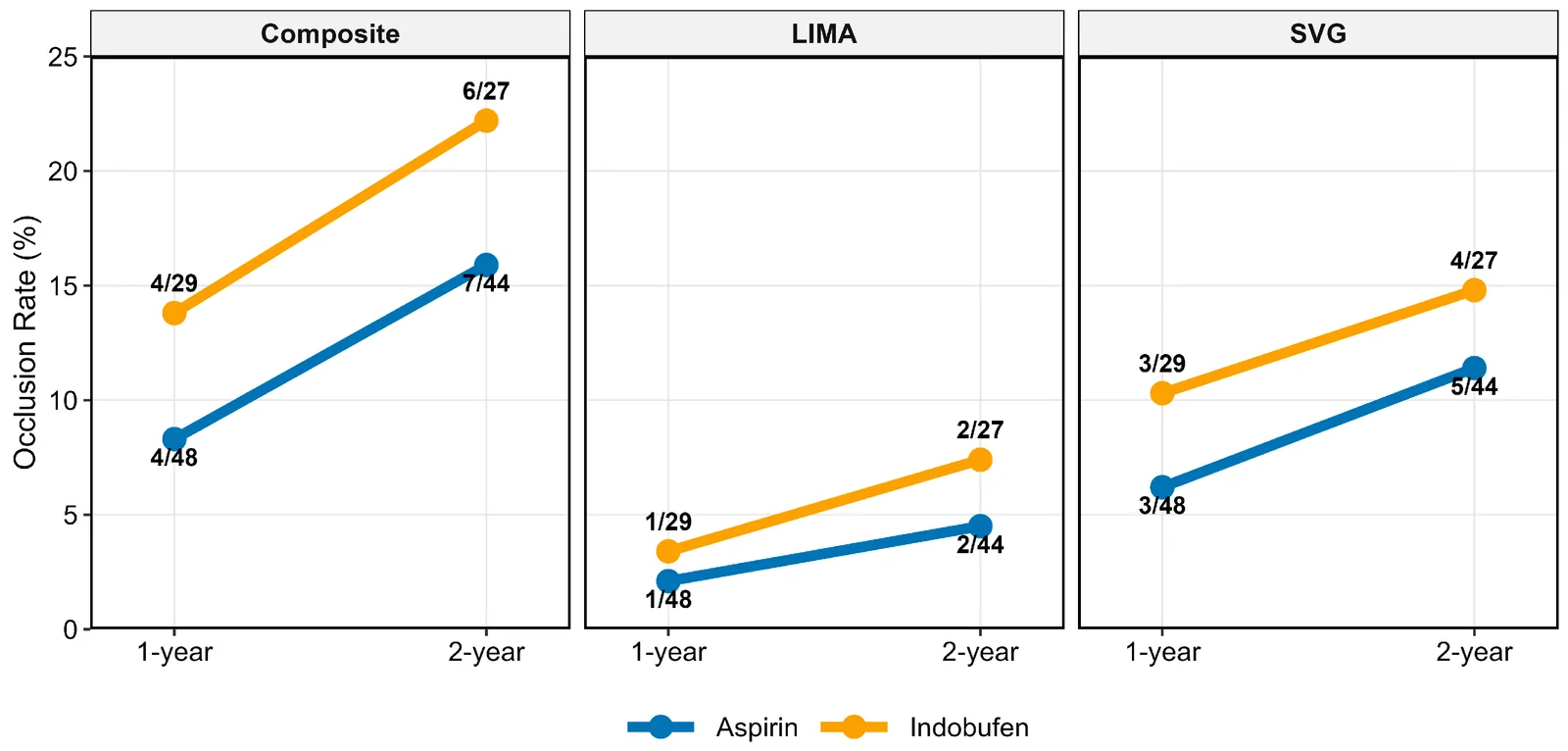
Descriptive graft-surveillance outcomes according to the initial discharge antiplatelet pathway. Outcomes are shown among patients with evaluable graft imaging at each time point. Graft imaging was performed during clinical follow-up and was not mandated by the study protocol; therefore, these findings should be interpreted descriptively rather than as comparative patency endpoints. LIMA, left internal mammary artery; SVG, saphenous vein graft.

Graft imaging was evaluable in 77 of 79 patients (97.5%) at 1 year and 71 of 79 patients (89.9%) at 2 years. At 2 years, imaging was evaluable in 44 of 49 patients (89.8%) in the aspirin-based pathway and 27 of 30 patients (90.0%) in the indobufen-based pathway. Two-year composite graft occlusion was observed in 7 of 44 evaluable aspirin-pathway patients (15.9%) and 6 of 27 evaluable indobufen-pathway patients (22.2%). These graft-surveillance findings are summarized in Table 3 and Figure 6 and should be interpreted descriptively because imaging was not mandated by the study protocol. Detailed graft imaging timing, modality, evaluability, and missingness are summarized in Supplementary Table S7.

## 4. Discussion

Postoperative antiplatelet therapy after CABG remains largely aspirin-centered; however, current guidelines increasingly recommend individualized treatment according to ischemic and bleeding risks [1,4,5,6]. For chronic coronary syndromes, lifelong low-dose aspirin remains the standard approach after CABG, whereas DAPT may be considered in selected patients with a higher risk of graft failure and a lower risk of bleeding [1,4,5]. For patients undergoing CABG after acute coronary syndrome, DAPT is generally resumed once postoperative hemostasis is considered adequate [6]. Nevertheless, CABG-specific randomized evidence remains limited, and evidence from PCI populations cannot be directly transferred to postoperative CABG care settings [4,5,6]. Recent studies have also highlighted the trade-off between graft protection and bleeding. Ticagrelor plus aspirin may reduce saphenous vein graft failure, but this benefit is accompanied by more clinically important bleeding [7,8]. Long-term DACAB follow-up suggested fewer major cardiovascular events with one year of ticagrelor-based DAPT in selected patients [9]. Taken together, these data support a net clinical benefit approach rather than escalation of antiplatelet intensity based on graft patency alone [4,7,8,9]. Although derived from a TAVR rather than CABG population, a recent Heart Surgery Forum study also emphasized the bleeding and transfusion burden associated with perioperative DAPT, reinforcing the broader need for individualized antiplatelet decisions in older cardiovascular patients [28].

Indobufen should be interpreted in this clinical context. Although it is not an established replacement for aspirin after CABG, its reversible cyclooxygenase inhibition and recovery of platelet function after discontinuation may be relevant in older surgical patients, especially when gastrointestinal vulnerability, bleeding concerns, temporary treatment interruption, or medication persistence affects postoperative management [17,18]. Recent Asian data provide useful context. Indobufen-based DAPT has been evaluated in Chinese PCI populations in the OPTION trial and ASPIRATION registry, and a recent elderly ACS PCI cohort further extended this question to older patients [21,22,23]. Systematic reviews have also summarized the broader evidence for indobufen in coronary artery disease, although these data are largely driven by non-CABG settings [19,20]. CABG-specific data remain limited to an earlier graft-patency study, a Chinese CABG randomized study, and a recent multicenter retrospective post-CABG cohort [24,25,26]. These studies support the clinical relevance of examining indobufen after CABG, but they do not justify interpreting the present findings as evidence of superiority, equivalence, noninferiority, or clinical interchangeability.

The present study was designed to reflect a practical clinical decision rather than a simple comparison of two isolated DAPT regimens [1,4,5,6]. Patients were classified according to a staged discharge pathway: DAPT during the first postoperative year, followed by maintenance therapy with the same cyclooxygenase inhibitor in the second year. This design is clinically relevant because older postoperative patients may not remain on the originally planned regimen if bleeding, gastrointestinal symptoms, drug access, outpatient physician decisions, or patient preference intervene [13,14,15]. After stabilizing IPTW, measured covariate balance improved, although residual imbalance remained for prior myocardial infarction and prior PCI. The 24-month clinical outcomes did not show statistically significant associations between the initial discharge pathway and MACCE, BARC grade ≥2 bleeding, or NACE. Because the estimates were imprecise and confidence intervals were wide, these findings should not be interpreted as evidence of superiority, equivalence, noninferiority, or interchangeability. They should instead be regarded as hypothesis-generating observations supporting further study of tolerability-aware antiplatelet pathways in bleeding-vulnerable older CABG patients [13,14,15].

The graft-surveillance results should also be interpreted cautiously. Graft imaging was performed during clinical follow-up and was not mandated by the study protocol. Findings were summarized descriptively among patients with evaluable imaging at each time point. Consequently, patients who died before the planned imaging, avoided contrast due to renal dysfunction, declined surveillance imaging, or underwent imaging outside the hospital may have been unevenly represented in the evaluable imaging cohort. These factors introduce survival-related and practice-related selection bias. Therefore, graft findings are best viewed as descriptive surveillance data rather than comparative evidence of graft patency differences between the two pathways.

Several unmeasured factors may have influenced treatment selection and persistence. In older adults after CABG, antiplatelet decisions are not solely determined by antithrombotic potency. Socioeconomic status, medication access, clinician preference, patient preference, postoperative discomfort, functional limitation, rehabilitation participation, gastrointestinal symptoms, and perceived bleeding risk may all affect adherence or pathway modification [13,14,15,29]. Formal frailty assessment was not routinely available in this retrospective cohort. Although albumin, hemoglobin, renal function, and left ventricular ejection fraction were included in the propensity-score model, these variables do not replace structured frailty assessment. These variables, together with frailty, functional status, cognition, and other geriatric domains, were not fully captured and may have contributed to residual confounding despite IPTW.

This study had some limitations. The sample size was small, and the number of clinical events was low, which limited the statistical power, especially for myocardial infarction, ischemic stroke, repeat revascularization, and major bleeding. IPTW improved the balance across measured baseline covariates but could not address unmeasured confounding. Treatment allocation was determined by treating physicians in routine practice, and the specific determinants of pathway selection were not systematically recorded. Patients with unavailable pathway information, missing key propensity-score covariates, or incomplete ascertainment of 24-month clinical outcomes were excluded, which may have introduced selection bias. Treatment modifications occurred during follow-up, and temporary interruptions, adherence, and all patient-level dose changes could not be captured with uniform completeness. Graft imaging was not protocol-defined and should not be treated as a formal comparative patency endpoint. Finally, this was a single-center Chinese cohort, and local prescribing patterns may limit its generalizability. Future prospective studies should include standardized bleeding assessments, adherence monitoring, recovery status evaluations, frailty or functional-status assessment, treatment interruption tracking, and protocol-defined graft imaging.

## 5. Conclusions

In this real-world cohort of patients aged 70 years and older undergoing CABG for unstable angina, an initial indobufen-based discharge pathway was not statistically significantly associated with 24-month MACCE, BARC grade ≥2 bleeding, or NACE compared with an aspirin-based pathway after stabilized IPTW. By evaluating a staged discharge strategy used in routine practice, this study adds CABG-specific data on indobufen in an older population in whom antiplatelet decisions often involve ischemic risk, bleeding vulnerability, gastrointestinal tolerance, and treatment persistence. These findings should be viewed as exploratory given the observational design, limited sample size, and potential residual confounding. Larger prospective studies with structured frailty assessment, adherence monitoring, and protocol-defined graft imaging are needed.

## Figures and Tables

**Table 1:** Baseline characteristics and covariate balance before and after inverse probability of treatment weighting.

Characteristic	Aspirin-Based (*n* = 49)	Indobufen-Based (*n* = 30)	Unweighted Absolute SMD	Weighted Absolute SMD
Age, years	74.1 ± 3.6	74.2 ± 3.5	0.033	0.079
Male sex	34/49 (69.4)	23/30 (76.7)	0.161	0.027
Prior myocardial infarction	8/49 (16.3)	5/30 (16.7)	0.009	0.129
Prior percutaneous coronary intervention	12/49 (24.5)	8/30 (26.7)	0.049	0.128
Prior gastrointestinal bleeding	2/49 (4.1)	3/30 (10.0)	0.242	0.059
Prior stroke	9/49 (18.4)	6/30 (20.0)	0.041	0.054
Diabetes mellitus	18/49 (36.7)	10/30 (33.3)	0.070	0.006
Current smoking	11/49 (22.4)	7/30 (23.3)	0.021	0.086
Proton-pump inhibitor use	17/49 (34.7)	17/30 (56.7)	0.449	0.014
Early postoperative bleeding	3/49 (6.1)	4/30 (13.3)	0.252	0.009
Hemoglobin, g/L	126.9 ± 12.9	126.5 ± 15.1	0.033	0.063
Creatinine, μmol/L	91.3 ± 15.3	93.8 ± 19.8	0.148	0.067
Albumin, g/L	37.8 ± 3.1	37.9 ± 3.6	0.039	0.075
Left ventricular ejection fraction, %	60.7 ± 7.1	60.9 ± 7.1	0.034	0.052
Number of grafts	3.0 (3.0–3.0)	3.0 (3.0–3.0)	0.061	0.022
Ticagrelor as P2Y12 inhibitor	8/49 (16.3)	4/30 (13.3)	0.082	0.059

Values are presented as mean ± standard deviation, median (interquartile range), or *n*/N (%), as appropriate. This table is restricted to the 16 prespecified baseline covariates included in the propensity-score model; therefore, unweighted and weighted standardized mean differences are reported only for these variables. Stabilized inverse probability of treatment weights were derived from the propensity-score model and were not truncated in the primary analysis. Additional baseline and procedural variables not included in the propensity-score model are summarized descriptively in Supplementary Table S3 without IPTW SMD columns. SMD, standardized mean difference; MI, myocardial infarction; PCI, percutaneous coronary intervention.

**Table 2:** Twenty-four-month clinical outcomes according to the discharge antiplatelet pathway.

Outcome	Aspirin- Based (*n* = 49)	Indobufen- Based (*n* = 30)	IPTW-Weighted Cox HR (95% CI)	*p*-Value
Main clinical effectiveness outcome				
MACCE	7/49 (14.3)	5/30 (16.7)	0.858 (0.257–2.866)	0.803
Additional clinical outcomes				
All-cause death	4/49 (8.2)	2/30 (6.7)	Descriptive only	—
Cardiovascular death	2/49 (4.1)	1/30 (3.3)	Descriptive only	—
Non-cardiovascular death	2/49 (4.1)	1/30 (3.3)	Descriptive only	—
nonfatal MI	3/49 (6.1)	1/30 (3.3)	Descriptive only	—
Ischemic stroke	2/49 (4.1)	1/30 (3.3)	Descriptive only	—
Clinically driven repeat revascularization	0/49 (0.0)	2/30 (6.7)	Not estimable	—
Safety outcomes				
BARC grade ≥2 bleeding	9/49 (18.4)	3/30 (10.0)	0.359 (0.093–1.385)	0.137
BARC grade ≥3 bleeding	2/49 (4.1)	1/30 (3.3)	Descriptive only	—
Net adverse clinical events				
NACE	15/49 (30.6)	7/30 (23.3)	0.526 (0.204–1.360)	0.185

Values are presented as *n*/N (%) unless otherwise indicated. Hazard ratios are for the indobufen-based pathway compared with the aspirin-based pathway. IPTW-weighted Cox hazard ratios are reported for the prespecified composite clinical outcomes when estimable. Component outcomes and cause-specific death categories are summarized descriptively because of the small number of events. Clinically driven repeat revascularization was not estimable because no events occurred in the aspirin-based pathway. A dash indicates that a *p*-value was not calculated. IPTW, inverse probability of treatment weighting; HR, hazard ratio; CI, confidence interval; MACCE, major adverse cardiovascular and cerebrovascular events; BARC, Bleeding Academic Research Consortium; NACE, net adverse clinical events.

**Table 3:** Descriptive fixed-time graft-surveillance outcomes according to the initial discharge antiplatelet pathway.

Outcome	Aspirin-Based Pathway	Indobufen-Based Pathway
One-year		
One-year graft imaging evaluable	48/49 (98.0)	29/30 (96.7)
Timing of one-year imaging, days after surgery, median (IQR)	371.5 (359.5–386.8)	364.0 (343.0–384.0)
One-year composite graft occlusion	4/48 (8.3)	4/29 (13.8)
One-year LIMA graft occlusion	1/48 (2.1)	1/29 (3.4)
One-year SVG occlusion	3/48 (6.2)	3/29 (10.3)
Two-year		
Two-year graft imaging evaluable	44/49 (89.8)	27/30 (90.0)
Timing of two-year imaging, days after surgery, median (IQR)	736.0 (708.0–750.2)	731.0 (715.5–761.0)
Two-year composite graft occlusion	7/44 (15.9)	6/27 (22.2)
Two-year LIMA graft occlusion	2/44 (4.5)	2/27 (7.4)
Two-year SVG occlusion	5/44 (11.4)	4/27 (14.8)

Values are presented as *n*/N (%) or median (interquartile range), as appropriate. Graft-surveillance outcomes were calculated among patients with evaluable graft imaging at each time point. Composite graft occlusion was defined at the patient level as any graft occlusion identified on annual coronary angiography or computed tomography angiography. LIMA and SVG occlusion were summarized separately. These analyses were descriptive because graft imaging was performed during clinical follow-up and was not mandated by the study protocol. LIMA, left internal mammary artery; SVG, saphenous vein graft; IQR, interquartile range.

## Data Availability

The data supporting the findings of this study are not publicly available due to privacy and ethical restrictions but are available from the corresponding author upon reasonable request.

## Supplementary Materials

The following supporting information can be downloaded at: https://www.mdpi.com/article/10.3390/jcdd13080356/s1, Table S1: Baseline characteristics of patients included in the final analytic cohort and those excluded because of incomplete ascertainment of 24-month clinical outcomes; Table S2: Detailed reasons for exclusion due to unavailable initial discharge antiplatelet pathway information and/or propensity-score covariates; Table S3: Additional baseline and procedural characteristics not included in the propensity-score model; Table S4A: Antiplatelet treatment details and DAPT initiation timing according to the initial discharge antiplatelet pathway; Table S4B: Documented cyclooxygenase-inhibitor pathway modifications during follow-up; Table S5: Clopidogrel-only sensitivity analysis; Table S6: Sensitivity analyses for 24-month clinical outcomes; Table S7: Graft imaging timing, modality, evaluability, and missingness; STROBE checklist.
