# Supplementary material for: Indobufen Versus Aspirin After CABG for Unstable Angina in Patients Aged 70 Years and Older
Source: J Cardiovasc Dev Dis. 2026 Jul 29;13(8):356. doi: 10.3390/jcdd13080356 (PMC13513141; doi:10.3390/jcdd13080356)
Supplement: Supplementary file 1 [file jcdd-13-00356-s001.zip › STROBE_Checklist_Cohort_jcdd-4354296.pdf]

# STROBE Statement—Checklist of Items That Should Be Included in Reports of Cohort Studies

## Completed for Manuscript ID jcdd-4354296

**Manuscript title** Indobufen Versus Aspirin After CABG for Unstable Angina in Patients Aged 70 Years and Older
**Study design** Single-center retrospective cohort study
**Checklist basis** Official STROBE cohort-study checklist; page references correspond to manuscript.v8(8) (15 pages, based on the pagination saved in the Word file).

| Section / topic | Item No. | Recommendation | Page(s) / location in manuscript |
|---|---|---|---|
| Title and abstract | 1(a) | Indicate the study's design with a commonly used term in the title or the abstract | Abstract—Methods, p. 1 (the design is stated as a single-center retrospective cohort study). |
| Title and abstract | 1(b) | Provide in the abstract an informative and balanced summary of what was done and what was found | Abstract, p. 1. |
| Introduction<br>Background/rationale | 2 | Explain the scientific background and rationale for the investigation being reported | Introduction, paragraphs 1–3, p. 2. |
| Introduction<br>Objectives | 3 | State specific objectives, including any prespecified hypotheses | Introduction, final paragraph, p. 2. |
| Methods<br>Study design | 4 | Present key elements of study design early in the paper | Methods 2.1, first paragraph, pp. 2–3. |
| Methods<br>Setting | 5 | Describe the setting, locations, and relevant dates, including periods of recruitment, exposure, follow-up, and data collection | Methods 2.1, pp. 2–3 (institutional registry/medical records; 1 January 2017–31 December 2021; fixed 24-month outcome ascertainment). |
| Methods<br>Participants | 6(a) | Give the eligibility criteria, and the sources and methods of selection of participants. Describe methods of follow-up | Methods 2.1, pp. 2–3, and 2.3–2.4, pp. 3–4; Results 3.1, pp. 4–5; Figure 1, p. 5. |
| Methods<br>Participants | 6(b) | For matched studies, give matching criteria and number of exposed and unexposed | Not applicable. The study used stabilized inverse probability of treatment weighting rather than matching. |
| Methods<br>Variables | 7 | Clearly define all outcomes, exposures, predictors, potential confounders, and effect modifiers. Give diagnostic criteria, if applicable | Methods 2.2, p. 3; Methods 2.4–2.5, pp. 3–4; Table 1, pp. 5–6. |
| Methods<br>Data sources/measurement | 8* | For each variable of interest, give sources of data and details of methods of assessment (measurement). Describe comparability of assessment methods if there is more than one group | Methods 2.1, pp. 2–3, and 2.4, pp. 3–4 (registry, medical records, follow-up documentation, and institutional/external imaging records). |

| Section / topic | Item No. | Recommendation | Page(s) / location in manuscript |
|---|---|---|---|
| Methods<br>Bias | 9 | Describe any efforts to address potential sources of bias | Methods 2.2, p. 3; Methods 2.4–2.5, pp. 3–4; Discussion, pp. 11–13. |
| Methods<br>Study size | 10 | Explain how the study size was arrived at | Methods 2.1, pp. 2–3; Results 3.1, pp. 4–5; Figure 1, p. 5 (prespecified study period and screened/excluded/included counts). |
| Methods<br>Quantitative variables | 11 | Explain how quantitative variables were handled in the analyses. If applicable, describe which groupings were chosen and why | Methods 2.5, p. 4; Table 1 and its footnote, pp. 5–6. |
| Methods<br>Statistical methods | 12(a) | Describe all statistical methods, including those used to control for confounding | Methods 2.5, p. 4 (propensity-score model, stabilized IPTW, Cox models, balance diagnostics, and sensitivity analyses). |
| Methods<br>Statistical methods | 12(b) | Describe any methods used to examine subgroups and interactions | Not applicable. No formal subgroup or interaction analyses were performed. The clopidogrel-only restricted analysis is reported as a sensitivity analysis under Items 12(e) and 17. |
| Methods<br>Statistical methods | 12(c) | Explain how missing data were addressed | Methods 2.3, p. 3, and 2.5, p. 4 (complete-case propensity-score cohort; no imputation); Supplementary Tables S1–S2 and S7. |
| Methods<br>Statistical methods | 12(d) | If applicable, explain how loss to follow-up was addressed | Methods 2.3–2.4, pp. 3–4; Results 3.1, pp. 4–5; Supplementary Table S1 (incomplete 24-month clinical ascertainment) and Table S7 (imaging evaluability/missingness). |
| Methods<br>Statistical methods | 12(e) | Describe any sensitivity analyses | Methods 2.2, p. 3, and 2.5, p. 4; Results 3.4, p. 7; Supplementary Tables S5–S6. |
| Results<br>Participants | 13(a)* | Report numbers of individuals at each stage of study—eg numbers potentially eligible, examined for eligibility, confirmed eligible, included in the study, completing follow-up, and analysed | Results 3.1, pp. 4–5; Figure 1, p. 5. |
| Results<br>Participants | 13(b)* | Give reasons for non-participation at each stage | Results 3.1, pp. 4–5; Figure 1, p. 5; Supplementary Table S2. |
| Results<br>Participants | 13(c)* | Consider use of a flow diagram | Figure 1, p. 5. |
| Results<br>Descriptive data | 14(a)* | Give characteristics of study participants (eg demographic, clinical, social) and information on exposures and potential confounders | Table 1, pp. 5–6; Supplementary Table S3. |
| Results<br>Descriptive data | 14(b)* | Indicate number of participants with missing data for each variable of interest | Methods 2.3, p. 3, and 2.5, p. 4; Results 3.1, pp. 4–5; Supplementary Tables S1–S2 and S7. |
| Results<br>Descriptive data | 14(c)* | Summarise follow-up time (eg, average and total amount) | Methods 2.1–2.4, pp. 2–4 (fixed 24-month clinical outcome window); Results 3.4–3.5, pp. 7–11. |
| Results<br>Outcome data | 15* | Report numbers of outcome events or summary measures over time | Table 2, pp. 7–8; Figures 3–5, pp. 8–9; Table 3, p. 10. |

| Section / topic | Item No. | Recommendation | Page(s) / location in manuscript |
|---|---|---|---|
| Results<br>Main results | 16(a) | Give unadjusted estimates and, if applicable, confounder-adjusted estimates and their precision (eg, 95% confidence interval). Make clear which confounders were adjusted for and why they were included | Methods 2.5, p. 4; Table 1, pp. 5–6; Table 2, pp. 7–8 (crude event counts/percentages and IPTW-weighted Cox HRs with 95% CIs). |
| Results<br>Main results | 16(b) | Report category boundaries when continuous variables were categorized | Not applicable. Continuous covariates were not categorized for the primary propensity-score or outcome models. |
| Results<br>Main results | 16(c) | If relevant, consider translating estimates of relative risk into absolute risk for a meaningful time period | Table 2, pp. 7–8 (absolute 24-month event counts and percentages are reported). |
| Results<br>Other analyses | 17 | Report other analyses done—eg analyses of subgroups and interactions, and sensitivity analyses | Results 3.4, p. 7; Supplementary Tables S5–S6. |
| Discussion<br>Key results | 18 | Summarise key results with reference to study objectives | Discussion, paragraphs 1–3, pp. 11–12; Conclusions, p. 13. |
| Discussion<br>Limitations | 19 | Discuss limitations of the study, taking into account sources of potential bias or imprecision. Discuss both direction and magnitude of any potential bias | Discussion, graft-surveillance bias paragraph, p. 12; limitations paragraph, pp. 12–13. |
| Discussion<br>Interpretation | 20 | Give a cautious overall interpretation of results considering objectives, limitations, multiplicity of analyses, results from similar studies, and other relevant evidence | Discussion, pp. 11–13; Conclusions, p. 13. |
| Discussion<br>Generalisability | 21 | Discuss the generalisability (external validity) of the study results | Discussion, limitations paragraph, pp. 12–13 (single-center Chinese cohort and local prescribing patterns). |
| Other information<br>Funding | 22 | Give the source of funding and the role of the funders for the present study and, if applicable, for the original study on which the present article is based | Funding statement, p. 13; funder-role statement in Conflicts of Interest, p. 14. |
