# Supplementary material for: Indobufen Versus Aspirin After CABG for Unstable Angina in Patients Aged 70 Years and Older
Source: J Cardiovasc Dev Dis. 2026 Jul 29;13(8):356. doi: 10.3390/jcdd13080356 (PMC13513141; doi:10.3390/jcdd13080356)
Supplement: Supplementary file 1 [file jcdd-13-00356-s001.zip › Supplementary_tableS1-S7.pdf]

Table S1. Baseline characteristics of patients included in the final analytic cohort and those excluded because of incomplete ascertainment of 24-month clinical outcomes.

**Characteristic**	**Included (*n* = 79)**	**Excluded (*n* = 79)**	***p*-value**	**SMD**
Age, years	73 (72–75)	75 (73–77)	0.016	0.265
Male sex	57/79 (72.2)	55/79 (69.6)	0.861	0.056
Body mass index, kg/m^2	23.7 (21–25.6)	24 (21–26)	0.974	0.068
Hypertension	55/79 (69.6)	54/79 (68.4)	1	0.027
Diabetes mellitus	28/79 (35.4)	32/79 (40.5)	0.623	0.104
Hyperlipidemia	11/79 (13.9)	11/79 (13.9)	1	0
Current smoking	18/79 (22.8)	20/79 (25.3)	0.853	0.059
Prior myocardial infarction	13/79 (16.5)	9/79 (11.4)	0.491	0.147
Prior stroke	15/79 (19.0)	16/79 (20.3)	1	0.032
=	5/79 (6.3)	5/79 (6.3)	1	0
Prior percutaneous coronary intervention	20/79 (25.3)	20/79 (25.3)	1	0
Proton-pump inhibitor use	34/79 (43.0)	38/79 (48.1)	0.632	0.102
Beta-blocker use	53/79 (67.1)	60/79 (75.9)	0.29	0.197
ACE inhibitor or ARB use	27/79 (34.2)	31/79 (39.2)	0.621	0.105
Left ventricular ejection fraction, %	62 (60–65)	60 (53.5–63)	0.022	0.247
Hemoglobin, g/L	128 (116.5–137)	128 (119.5–135)	0.956	0.054
Creatinine, μmol/L	93 (83–102.5)	89 (79–100)	0.363	0.162
Chronic kidney disease	8/79 (10.1)	11/79 (13.9)	0.626	0.117
Baseline anemia	38/79 (48.1)	48/79 (60.8)	0.15	0.256
Number of grafts	3 (3–3)	3 (3–3)	0.839	0.02
Graft complexity score	3 (2–3)	3 (2–3)	0.33	0.16
Perioperative transfusion, units	0 (0–0)	0 (0–0)	0.355	0.068
Left main disease	42/79 (53.2)	42/79 (53.2)	1	0
On-pump CABG	12/79 (15.2)	11/79 (13.9)	1	0.036
Early postoperative bleeding	7/79 (8.9)	11/79 (13.9)	0.453	0.16
Ticagrelor as P2Y12 inhibitor	12/79 (15.2)	16/79 (20.3)	0.533	0.133

Values are presented as median (interquartile range) or n/N (%), as appropriate. This table compares patients included in the final analytic cohort with patients excluded because 24-month clinical outcome ascertainment was incomplete. The comparison was performed to evaluate potential selection bias related to outcome ascertainment. Standardized mean differences are provided to describe the magnitude of between-group differences and should not be interpreted as IPTW balance diagnostics. The term "incomplete ascertainment of 24-month clinical outcomes" was used rather than "lost to follow-up" because incomplete ascertainment may reflect heterogeneous reasons in this retrospective cohort. SMD, standardized mean difference; ARB, angiotensin receptor blocker; ACE, angiotensin-converting enzyme; CABG, coronary artery bypass grafting; LVEF, left ventricular ejection fraction; MI, myocardial infarction; PCI, percutaneous coronary intervention.

Table S2. Detailed reasons for exclusion due to unavailable initial discharge antiplatelet pathway information and/or propensity-score covariates.

Initial discharge antiplatelet pathway could not be reliably determined	18	56.3
Pathway known but key propensity-score covariates unavailable	14	43.7
Total	32	100

Values are presented as n and percentage. These exclusions correspond to the 32 patients removed because the initial discharge antiplatelet pathway could not be reliably determined or because key propensity-score covariates were unavailable. The two categories were applied after exclusion of redo or concomitant procedures and oral anticoagulation or non-concordant discharge antithrombotic regimens. PS, propensity score.

Table S3. Additional baseline and procedural characteristics not included in the propensity-score model.

Characteristic	Aspirin-based pathway (*n* = 49)	Indobufen-based pathway (*n* = 30)
Body mass index, kg/m²	23.7 ± 3.2	23.7 ± 4.1
Hypertension	35/49 (71.4)	20/30 (66.7)
Hyperlipidemia	7/49 (14.3)	4/30 (13.3)
Chronic kidney disease	4/49 (8.2)	4/30 (13.3)
Baseline anemia	24/49 (49.0)	14/30 (46.7)
Beta-blocker use	31/49 (63.3)	22/30 (73.3)
ACE inhibitor or ARB use	18/49 (36.7)	9/30 (30.0)
LDL cholesterol, mmol/L	2.01 ± 0.92	1.76 ± 0.51
eGFR, mL/min/1.73 m²	76.8 ± 15.2	77.3 ± 16.5
Perioperative transfusion, units	0.0 (0.0–0.0)	0.0 (0.0–0.0)
Graft complexity score	3.0 (2.0–3.0)	2.5 (2.0–3.0)
Left main disease	27/49 (55.1)	15/30 (50.0)
On-pump CABG	7/49 (14.3)	5/30 (16.7)
ICU stay, days	2.4 (2.1–2.9)	2.2 (2.0–2.6)
Postoperative hospital stay, days	8.7 (7.7–9.6)	8.4 (7.8–8.8)

Values are presented as mean ± standard deviation, median (interquartile range), or n/N (%), as appropriate. These variables are shown for clinical description and were not included in the final propensity-score model. Therefore, IPTW-weighted standardized mean differences are not reported for this table. ACE, angiotensin-converting enzyme; ARB, angiotensin receptor blocker; LDL, low-density lipoprotein; eGFR, estimated glomerular filtration rate; CABG, coronary artery bypass grafting; ICU, intensive care unit.

Table S4A. Antiplatelet treatment details and DAPT initiation timing according to the initial discharge antiplatelet pathway.

Treatment detail	Aspirin-based pathway (*n* = 49)	Indobufen-based pathway (*n* = 30)
Initial aspirin 100 mg once daily	49/49 (100.0)	0/30 (0.0)
Initial indobufen 100 mg twice daily	0/49 (0.0)	30/30 (100.0)
Clopidogrel as background P2Y12 inhibitor	41/49 (83.7)	26/30 (86.7)
Ticagrelor as background P2Y12 inhibitor	8/49 (16.3)	4/30 (13.3)
DAPT start 6–12 h after surgery	12/49 (24.5)	7/30 (23.3)
DAPT start >12–24 h after surgery	27/49 (55.1)	18/30 (60.0)
DAPT start >24–48 h after surgery	10/49 (20.4)	5/30 (16.7)
Planned DAPT duration 12 months	49/49 (100.0)	30/30 (100.0)
Actual DAPT duration, months	12.0 (11.0–12.0)	12.0 (11.2–12.0)

Values are presented as n/N (%) or median (interquartile range), as appropriate. The initial discharge antiplatelet pathway was defined according to the cyclooxygenase-inhibitor component prescribed at discharge. DAPT, dual antiplatelet therapy; P2Y12, P2Y12 receptor inhibitor.

Table S4B. Documented cyclooxygenase-inhibitor pathway modifications during follow-up.

Case	Initial pathway	Direction	Day after discharge	Follow-up phase	Documented reason
1	Indobufen	Indobufen to aspirin	96	year 1 DAPT phase	cost/access
2	Aspirin	Aspirin to indobufen	110	year 1 DAPT phase	GI intolerance/dyspepsia
3	Aspirin	Aspirin to indobufen	188	year 1 DAPT phase	BARC 2 bleeding
4	Indobufen	Indobufen to aspirin	221	year 1 DAPT phase	formulary change
5	Aspirin	Aspirin to indobufen	274	year 1 DAPT phase	recurrent gastric symptoms
6	Indobufen	Indobufen to aspirin	365	at completion of year 1	patient preference after year 1
7	Aspirin	Aspirin to indobufen	402	year 2 SAPT phase	physician preference after year 1

Pathway modification refers to a documented change in the cyclooxygenase-inhibitor component from aspirin to indobufen or from indobufen to aspirin during follow-up. The primary analysis classified patients according to the initial discharge pathway. Temporary treatment interruptions, day-to-day adherence, and all patient-level dose changes were not captured with uniform completeness.

Table S5. Clopidogrel-only sensitivity analysis.

Outcome	Aspirin-based pathway	Indobufen-based pathway	IPTW-adjusted odds ratio (95% CI)	P value
Major adverse cardiovascular and cerebrovascular events	6/41 (14.6)	4/26 (15.4)	0.99 (0.24-4.12)	0.985
All-cause death	4/41 (9.8)	2/26 (7.7)	0.72 (0.11-4.53)	0.725
BARC grade >=2 bleeding	7/41 (17.1)	3/26 (11.5)	0.49 (0.11-2.15)	0.346
BARC grade >=3 bleeding	2/41 (4.9)	1/26 (3.8)	0.52 (0.04-6.15)	0.603
Net adverse clinical events	12/41 (29.3)	6/26 (23.1)	0.65 (0.20-2.12)	0.477

Table S6. Sensitivity analyses for 24-month clinical outcomes.

Section	Outcome	IPTW-weighted hazard ratio (95% CI)	*p*-value
A. Sensitivity analysis censoring at documented pathway modification	Major adverse cardiovascular and cerebrovascular events	0.679 (0.186-2.479)	0.5582
	All-cause death	NA (NA-NA)	NA
	BARC grade≥2 bleeding	0.359 (0.093-1.385)	0.1371
	Net adverse clinical events	0.760 (0.340-1.700)	0.504
B. Primary stabilized IPTW-weighted Cox analysis shown for comparison	Major adverse cardiovascular and cerebrovascular events	0.858 (0.257-2.866)	0.8032
	All-cause death	NA (NA-NA)	NA
	BARC grade ≥2 bleeding	0.359 (0.093-1.385)	0.1371
	Net adverse clinical events	0.526 (0.204-1.360)	0.1851
C. Sensitivity analysis using stabilized IPTW truncated at the 5th and 95th percentiles	Major adverse cardiovascular and cerebrovascular events	0.912 (0.279–2.979)	0.8783
	All-cause death	NA	NA
	BARC grade ≥2 bleeding	0.388 (0.102–1.476)	0.1649
	Net adverse clinical events	0.862 (0.397–1.870)	0.7063
D. Stabilized IPTW-weighted Cox analysis with additional adjustment for prior myocardial infarction and prior percutaneous coronary intervention	Major adverse cardiovascular and cerebrovascular events	0.765 (0.196–2.993)	0.7007
	All-cause death	NA	NA
	BARC grade ≥2 bleeding	0.358 (0.095–1.348)	0.1288
	Net adverse clinical events	0.503 (0.186–1.359)	0.1752

Section A presents a sensitivity analysis in which patients were censored at the time of documented pathway modification. Section B reproduces the primary stabilized IPTW-weighted Cox estimates for comparison. Section C uses stabilized weights truncated at the empirical 5th and 95th percentiles. Section D uses the original stabilized weights with additional adjustment for prior myocardial infarction and prior percutaneous coronary intervention. HR, hazard ratio; CI, confidence interval; IPTW, inverse probability of treatment weighting; BARC, Bleeding Academic Research Consortium; MACCE, major adverse cardiovascular and cerebrovascular events; NACE, net adverse clinical events; NE, not estimable.

Table S7. Graft imaging timing, modality, evaluability, and missingness.

Imaging detail	Aspirin-based pathway	Indobufen-based pathway
One-year		
Evaluable imaging	48/49 (98.0)	29/30 (96.7)
CTA among evaluable imaging	41/48 (85.4)	23/29 (79.3)
ICA among evaluable imaging	7/48 (14.6)	6/29 (20.7)
Timing, days after surgery	371.5 (359.5–386.8)	364.0 (343.0–384.0)
Two-year		
Evaluable imaging	44/49 (89.8)	27/30 (90.0)
CTA among evaluable imaging	37/44 (84.1)	21/27 (77.8)
ICA among evaluable imaging	7/44 (15.9)	6/27 (22.2)
Timing, days after surgery	736.0 (708.0–750.2)	731.0 (715.5–761.0)

Values are presented as n/N (%) or median (interquartile range), as appropriate. Graft imaging included coronary computed tomography angiography or invasive coronary angiography performed during clinical follow-up. Imaging was not mandated by the study protocol. Missing imaging should not be assumed to indicate absence of graft occlusion. CTA, computed tomography angiography; ICA, invasive coronary angiography; IQR, interquartile range.
